# Association analysis of germline mutations in CHEK2, PALB2, NBN and RECQL with the risk of ductal carcinoma in situ in Polish women

**DOI:** 10.1186/s13053-025-00320-z

**Published:** 2025-08-06

**Authors:** Sylwia Feszak, Wojciech Kluźniak, Igor Feszak, Magdalena Chady, Dominika Wokołorczyk, Klaudia Stempa, Helena Rudnicka, Katarzyna Gliniewicz, Anna Jakubowska, Marcin Lener, Maciej Czepukowicz, Tomasz Huzarski, Tadeusz Dębniak, Jacek Gronwald, Jan Lubiński, Cezary Cybulski

**Affiliations:** 1 Department of Genetics and Pathology, International Hereditary Cancer Center, Pomeranian Medical University in Szczecin, Szczecin, 71-252 Poland; 2 Department of Pediatrics, Pediatric Oncology, and Immunology, Pomeranian Medical University, Szczecin, 71-252 Poland; 3 Institute of Health Sciences, Pomeranian University in Słupsk, Słupsk, 76-200 Poland; 4 Department of Clinical Genetics and Pathology, University of Zielona Góra, Zielona Góra, 65-046 Poland

**Keywords:** CHEK, Breast cancer, Ductal carcinoma in situ, Poland, Germline mutations

## Abstract

**Background:**

The genetic background of ductal carcinoma in situ (DCIS) has not been well explored. Previously, we reported that Polish founder mutations of *BRCA1/2* confer susceptibility to DCIS. The aim of our study was to investigate the role of *CHEK2*, *PALB2*, *NBN* and *RECQL* mutations in the ethology of DCIS.

**Methods:**

We studied 564 Polish women with DCIS for eight Polish founder alleles, including four in *CHEK2* (c.1100delC, c.444 + 1G > A, del5395 and c.470T > C), two in *PALB2* (c.509_510delGA and c.172_175delTTGT), one in *NBN* (c.657_661delACAAA) and one in *RECQL* (c.1667_1667 + 3delAGTA). To investigate the association of these alleles with DCIS risk, we used mutation frequencies in cancer-free controls as a reference (4000 to 4702 controls for different variants). To analyze survival, patients were followed on average for 156 months.

**Results:**

A *CHEK2* mutation (all variants combined) was associated with an increased risk of DCIS (OR = 1.7, *p* = 0.003). The risk was higher for *CHEK2* truncating mutations (OR = 3.0, *p* = 0.001) than for a missense variant c.470T > C (OR = 1.5, *p* = 0.04). The risk was highest for carriers of *CHEK2* truncating mutations with a family history of breast cancer (OR = 4.2, *p* = 0.01). There were no deaths reported in 52 *CHEK2* mutation carriers during the follow up time. *PALB2*, *NBN* and *RECQL* mutations were rare among cases and were not associated with DCIS risk in Polish women.

**Conclusions:**

Based on the current study, women with a *CHEK2* mutation face an increased risk of DCIS. The presence of DCIS should be considered during surveillance of *CHEK2* mutation carriers. On the other hand, DCIS patients should receive genetic counseling and testing for *CHEK2* mutations.

## Background

Breast cancer is the most common malignancy in women, annually diagnosed in above two million women worldwide, including over twenty thousand cases in Poland [1, 2]. The genetic basis of breast cancer has been deeply explored over the several past decades [3–7]. The two major highly penetrant genes for breast cancer, *BRCA1* and *BRCA2*, were identified in 1994 and 1995, respectively [8, 9]. Since that, many other genes were reported to be associated with a genetic susceptibility to this cancer including *PALB2*, *CHEK2*, *ATM*,* BARD1*,* BLM*,* NBN*,* XRCC2*,* RECQL*,* RAD50*,* RAD51C*,* RAD51D*,* TP53*,* PTEN*,* NF1*,* STK11* and *CDH1* [10–17]. Now, identifying mutations in breast cancer susceptibility genes is used in clinical practice for risk prediction, prevention and surveillance in unaffected individuals and treatment of cancer patients [18–21].

The genetic background of invasive breast cancer has been well explored, but genetic determinants of non-invasive breast cancers have not been extensively studied [4, 22, 23]. Non-invasive breast tumors constitute about 10–15% of all breast cancers [24]. Ductal carcinoma in situ (DCIS) is the most common type of non-invasive breast cancer [25–27]. It represents about 90% of all non-invasive breast tumors [28, 29]. Most DCIS tumors are asymptomatic, identified by screening mammography [24]. DCIS is associated with a favorable prognosis, but it is a precursor of invasive ductal breast cancer, so it is important to identify risk factors for this disease as well [30, 31]. The incidence of DCIS varies significantly by a population. It has been reported that DCIS constitutes 5–25% of all breast cancer cases, and the lifetime risk of DCIS ranges between 0.5% and 3% in different countries [31]. These differences are explained by the fact that the breast screening programs vary in their recommendations and effectiveness across different populations [24].

According to Polish National Cancer Registry report, in year 2022 invasive breast cancer (C50 in ICD-10 classification) was diagnosed among 21.6 thousand women (cumulative risk of 10%), while in situ breast cancer (D05 in ICD-10 classification) was diagnosed in 1.43 thousand women (cumulative risk of 0.6%). Based on the report, DCIS constitutes about 5% of breast cancers in Poland, and the lifetime risk of DCIS is only approximately 0.5% in Polish women [32]. Given that the incidence of DCIS determined by a large meta-analysis of 13 post-mortem studies in women is as high as 9%, DCIS appears to be underdiagnosed both in Poland and elsewhere [33].

In 2019, we have conducted a comprehensive analysis of breast cancer susceptibility in Poland. We detected pathogenic mutations in 512 of 1,018 (50%) families with hereditary breast cancer (HBC), and we have found that 20 founder mutations in six genes, *BRCA1*, *BRCA2*, *CHEK2*, *PALB2*, *NBN* and *RECQL* are responsible for 82% of all Polish HBC families with detected mutations [34]. It is interesting to establish whether this panel of founder mutations in *BRCA1*, *BRCA2*, *CHEK2*, *PALB2*, *NBN* and *RECQL* contribute to DCIS burden in Poland.

To answer this question, previously we investigated if *BRCA1*/2 mutations predispose to DCIS. We found that *BRCA1*/2 founder mutations are responsible for 3% of DCIS cases in Poland. *BRCA2* variants conferred higher DCIS risk (OR = 11.3, *p* < 0.0001) than *BRCA1* mutations (OR = 3.3, *p* = 0.01) [35]. In the current study, we established whether mutations in non-*BRCA1*/2 genes (from the panel of 20 Polish founder alleles) predispose to DCIS. We analyzed the frequencies of 8 founder alleles in four genes including *CHEK2* (c.1100delC, c.444 + 1G > A, del5395 and c.470T > C), *PALB2* (c.509_510delGA and c.172_175delTTGT), *NBN* (c.657_661delACAAA) and *RECQL* (c.1667_1667 + 3delAGTA) among 564 Polish women with DCIS and compared with mutation frequencies in Polish cancer-free controls (4000 to 4702 controls for different variants). Only *CHEK2* mutations predisposed to DCIS, therefore we investigated their influence on age of DCIS diagnosis, the impact on survival among patients, and the effect of positive family history of breast cancer in *CHEK2* carriers on the risk of DCIS.

## Methods

### Patients

We studied a series of 564 women with ductal carcinoma in situ, diagnosed between 1997 and 2019, unselected for age and family history. The patients were derived from a group of 17,086 breast cancer cases registered at the Hereditary Cancer Center in Szczecin. We excluded patients with a personal history of breast cancer or ovarian cancer before diagnosis of DCIS. The diagnosis of DCIS, with or without micro-invasion, was confirmed by a pathology report in all subjects. Each patient provided a blood sample for DNA isolation within 2 years from the date of diagnosis.

All patients signed an informed consent for genetic testing. Each patient was self- or physician-referred for genetic counseling. Clinical, demographic, and self-reported ancestry information were obtained at the time of the interview. All women were Poles. A pedigree including cancer cases in first- and second-degree relatives and their ages of diagnosis was collected for each participant. Survival data (alive or dead; if deceased, the date of death) were obtained from the Polish Ministry of the Interior and Administration in February 2020. The study was approved by the Ethics Committee of Pomeranian Medical University in Szczecin (IRB No. KB-0012/34/05/2020/Z).

The mean age of diagnosis of DCIS among the 564 women was 55.3 years (median 55.0, range 23–91 years, SD 10.8); 27.3% (154/564) women were diagnosed with DCIS before the age 50 years, 7.1% (40/564) women were diagnosed before the age 40 years. In 145 (25.7%) cases, there was a family history of breast cancer in at least one first or second degree relative (familial cases). The median follow up was 156 months. Eighteen (3.2%) patients died within the follow-up. The cases were described in more detail previously [35].

### Mutation analysis

All 564 patients were tested for 8 mutations, including four in *CHEK2*; c.1100delC (p.Thr410Metfs), c.444 + 1G > A, del5395 (p.Thr410Metfs) and c.470T > C (p.Ile157Thr), two in *PALB2*: c.509_510delGA (p.Arg170Ilefs) and c.172_175delTTGT (p.Gln60Argfs), one allele of *NBN*: c.657_661delACAAA (p.Lys219Asnfs) and one in *RECQL*: c.1667_1667 + 3delAGTA (p.K555delinsMYKLIHYSFR) [36].

A large deletion of exon 9 and 10 of *CHEK2* (del5395) was detected by multiplex-PCR using two primer pairs; the first pair flanked breakpoint site in intron 8 of *CHEK2*; the second flanked breakpoint site in intron 10. In mutation negative cases, two PCR fragments of 379 and 522 bp were amplified from the wild type allele. In mutation positive cases the forward primer of the first pair and the reverse primer of the second pair amplified an additional fragment of 450 bp, as described previously [37].

The other 7 mutations: *CHEK2* c.1100delC, c.444 + 1G > A, c.470T > C; *PALB2* c.509_510delGA and c.172_175delTTGT; *NBN* c.657_661delACAAA and *RECQL* c.1667_1667 + 3delAGTA were genotyped in an automated thermal cycler (LightCycler^®^ Real-Time PCR 480 System). The 5 µl reaction mixture contained: 1 µl (10ng) of genomic DNA, 0.125 µl TaqMan Assay 40x (Thermo Fisher Scientific, Waltham, MA, USA), 2.5 µl of reaction mix (GoTaq^®^Probe qPCR Master Mix 2x, Promega), supplemented to 5 µl with 1,375 µl nuclease-free water. TaqMan assays are described in Table 1. Real-time PCR conditions were described previously [35].

**Table 1:** TaqMan assays used to detect Polish founder mutations of *CHEK2*, *PALB2*, *NBN* and *RECQL*

Assay ID	HGVS	dbSNP
Pre-designed assays		
C_34306823_20	NM_007194.4 (*CHEK2*): c.470T > C	rs17879961
C_336684713_10	NM_024675.4 (*PALB2*): c.509_510delGA	rs515726123
C_190888305_10	NM_024675.4 (*PALB2*): c.172_175delTTGT	rs180177173
C_203097466_10	NM_002485.5 (*NBN*): c.657_661delACAAA	rs587776650
C_324203460_10	NM_002907.4 (*RECQL*): c.1667_1667 + 3delAGTA	rs564485792
Custom-design assays		
Not available	NM_007194.4 (*CHEK2*): c.1100delC	rs555607708
Not available	NM_007194.4 (*CHEK2*): c.444 + 1G > A	rs121908698

### Statistical analysis

The prevalence of the 8 recurrent mutations was estimated in 564 DCIS patients. The odds ratios for DCIS given *CHEK2*, *PALB2*, *NBN* and *RECQL* status (positive versus negative) were calculated by two-by-two tables, using as a reference mutation frequencies in Polish cancer-free controls from our four previous studies [10, 14, 16, 38]. Additionally, we calculated odds ratios separately for each variant. Statistical significance (*p*-value) was assessed using Fisher exact test. All cases and controls were ethnic Poles.

To estimate all cause survival of women with DCIS, with and without a *CHEK2* mutation (other mutations were not considered because were rare), we followed DCIS patients from the date of diagnosis until the date of death or February 2020, if alive. The actuarial survival between *CHEK2* mutation carriers with DCIS and non-carriers with DCIS was compared, and *p*-value for difference was calculated using Cox regression analysis. Medians were compared using the Mann-Whitney test. Analyses were performed by MedCalc Version 22.014.

## Results

In this study of 564 women with DCIS, 57 (10.1%) carried a founder non-BRCA mutation in one of the three genes including variants in *CHEK2* (c.1100delC, c.444 + 1G > A, del5395 and c.470T > C), *PALB2* (c.509_510delGA) and *NBN* (c.657_661delACAAA). *CHEK2* mutations were the most common, detected in 52 (9.2%) women with DCIS. A *CHEK2* truncating mutation was detected in 37 (2.5%) patients and *CHEK2* c.470T > C missense variant was identified in 40 patients (7.1%). Two women carried two different *CHEK2* mutations (one carried c.1100delC and c.470T > C and the second had del5395 and c.470T > C). Mutations in *PALB2* and *NBN* were seen in 1 (0.2%) and 4 (0.7%) patients, respectively. *RECQL* c.1667_1667 + 3delAGTA allele was not observed among cases.

We investigated the association of the 8 founder mutations with DCIS, using as a reference allele frequencies in cancer-free controls from our previous studies [10, 14, 16, 38]. We saw a strong association of *CHEK2* mutations (all variants combined) with DCIS risk (OR = 1.7, 95%CI 1.2–2.3, *p* = 0.003). The odds ratio was higher (OR = 3.0, 95%CI 1.5–5.7, *p* = 0.001) for *CHEK2* truncating mutations (c444 + 1G > A, c.1100delC or del5395) than for a missense variant c.470T > C (OR = 1.5, 95%CI 1.05–2.11, *p* = 0.04). *PALB2*, *NBN* and *RECQL* mutations were not associated with the risk of DCIS (*p*–values between 0.6 and 1.0), and were not further considered (Table 2).

**Table 2:** Odds ratios for DCIS for Polish founder variants in *CHEK2*, *PALB2*, *NBN* and *RECQL* with corresponding *p*-values

Gene	Mutation	Cases *N* = 564		Controls^a^			OR	95%CI	*P*-value
		Positive	%	Positive	Total	%			
*CHEK2*	c444 + 1G > A	7	1.2	14	4346	0.3	3.9	1.3–10.3	0.007
	c.1100delC	1	0.2	7	4346	0.2	1.1	0.02–8.6	1.0
	del5395	6	1.1	16	4346	0.4	2.9	0.9–7.9	0.03
	Any truncating mutation^b^	14	2.5	37	4346	0.9	3.0	1.5–5.7	0.001
	c.470T > C missense variant	40	7.1	215	4346	4.9	1.5	1.05–2.1	0.04
	Any *CHEK2* mutation^c^	52	9.2	252	4346	5.8	1.7	1.2–2.3	0.003
*PALB2*	c.509_510delGA	1	0.2	7	4702	0.1	1.2	0.03–9.3	0.6
	c.172_175delTTGT	0	0.0	3	4702	0.1	-	-	1.0
	Any *PALB2* mutation^d^	1	0.2	10	4702	0.2	0.8	0.2–5.9	1.0
*NBN*	c.657_661delACAAA	4	0.7	22	4000	0.6	1.3	0.3–3.8	0.6
*RECQL*	c.1667_1667 + 3del AGTA	0	0.0	2	4702	0.04	-	-	1.0

^a^Mutation frequencies in 4000 to 4702 Polish cancer-free controls (for different variants) were derived from our previous studies [10, 14, 16, 38]

^b^c444+1G>A or c.1100delC or del5395

^c^c444+1G>A or c.1100delC or del5395 or c.470T>C

^d^c.509_510delGA or c.172_175delTTGT

We also investigated the influence of *CHEK2* mutations on the risk of early onset DCIS (age 50 years or below) and late onset disease (above age 50 years). The odds ratio for early onset DCIS given a *CHEK2* mutation (all variants combined) was 2.4 (95%CI 1.5–3.8) and was very significant (*p* = 0.0004). In contrast, the odds ratio for late onset disease was 1.4 (95%CI 0.9–2.1) and it was not statistically significant (*p* = 0.1) (Table 3). We also checked the effect of a positive family history of breast cancer on DCIS risk among *CHEK2* mutation carriers. The odds ratio for a *CHEK2* mutation was higher for cases with a positive family history (OR = 2.3, 95%CI 1.3–3.9, *p* = 0.003) than those with no family history of breast cancer (OR = 1.4, 95%CI 0.96–2.1, *p* = 0.06) (Table 3). The odds ratio was the highest for women with a *CHEK2* truncating mutation and family history of breast cancer (OR = 4.2, 95%CI 1.3–10.8, *p* = 0.01).

**Table 3:** Odds ratios for DCIS for *CHEK2* mutations by age of diagnosis and by family history of breast cancer

Group	Total No.	CHEK2 c444 + 1G > A		CHEK2 1100delC		CHEK2 del5395		Any CHEK2 truncating mutation^b^		CHEK2 c.470T > C missense variant		Any CHEK2 mutation^c^		OR	95%CI	*P*-value
		No.	%	No.	%	No.	%	No.	%	No.	%	No.	%			
All DCIS patients	564	7	1.2	1	0.2	6	1.1	14	2.5	40	7.1	52	9.2	1.6	1.2–2.3	0.003
Age ≤ 50 years	186	3	1.6	1	0.5	0	0.0	4	2.2	20	10.8	23	12.4	2.3	1.4–3.6	0.0008
Age > 50 years	378	4	1.1	0	0.0	6	1.6	10	2.6	20	5.3	29	7.7	1.3	0.9-2.0	0.14
Cases with FH^a^	145	3	2.0	1	0.7	1	0.7	5	3.4	13	9.0	18	12.4	2.3	1.3–3.9	0.003
Cases without FH^a^	419	4	1.0	0	0.0	5	1.2	9	2.1	27	6.4	34	8.1	1.4	0.96–2.1	0.06
Cancer-free controls	4346							37	0.9	215	4.9	252	5.8	ref	ref	ref

^a^FH - positive family history of breast cancer in first- or second-degree relative

^b^c444 + 1G > A or c.1100delC or del5395

^c^c444 + 1G > A or c.1100delC or del5395 or c.470T > C

Finally, we explored the impact of *CHEK2* mutations on survival of DCIS patients. The median follow-up among 564 women with DCIS was 156 months (range 1–270, SD 62.7), including 166 months (range 18–258, SD 54.6) for *CHEK2* mutation carriers versus 156 (range 1–270, SD 63.4) for non-carriers (*p* = 0.3, Mann-Whitney test). There were no deaths in 52 mutation carriers (0%) versus 18 deaths in 512 non-carriers (3.5%) during the follow up time (*p* = 0.4). The 10-year survival was 100% for carriers compared to 97% for non-carriers. The age-adjusted p-value for mortality associated with a *CHEK2* mutation was 0.9 (Cox regression analysis). There were no deaths among mutation carriers, so the hazard ratio (carriers vs. non-carriers) was not estimated.

## Discussion

In Poland, we have initiated a program to detect DNA mutations which predispose to DCIS. We focused on Polish founder mutations in *BRCA1*, *BRCA2*, *PALB2*, *CHEK2*, *NBN* and *RECQL* (18 alleles), which account for one-half of Polish breast cancer families [34]. Previously, we reported that Polish founder mutations of *BRCA1*/2 confer susceptibility to DCIS, and are responsible for 3% of DCIS cases in Poland [35]. In the current study, we focused on 8 recurrent mutations in four non-*BRCA1*/2 genes (from the panel of 20 Polish alleles) including *CHEK2* (c.1100delC, c.444 + 1G > A, del5395 and c.470T > C), *PALB2* (c.509_510delGA, c.172_175delTTGT), *NBN* (c.657_661delACAAA) and *RECQL* (c.1667_1667 + 3delAGTA).

We found a strong association of *CHEK2* mutations with an increased risk of DCIS. *CHEK2* founder mutations are present with a frequency of 6% in a genetically homogenous population of Poland [34]. According to our current study, *CHEK2* mutations (all alleles together) confer a 1.7-fold increased risk of DCIS. Based on these figures, we estimate that *CHEK2* founder mutations account for 4% of DCIS cases in Poland (population attributable risk estimate). We consider that *CHEK2* mutations may be important cause of DCIS in other homogenous populations, in which founder alleles of this gene are present, i.e. the Netherlands, Finland and Eastern European countries [39, 40]. Our results suggest that *CHEK2* truncating mutations confer a 3-fold (*p* = 0.001) increased risk of DCIS and a missense c.470T > C variant confers lower risk (OR = 1.5, *p* = 0.04).

In the current study, we observed strong association of *CHEK2* mutations (all variants combined) with cases diagnosed at age 50 or below (OR = 2.4, *p* = 0.0004), but not with cases diagnosed above age 50 (OR = 1.4, *p* = 0.1). Therefore, *CHEK2* mutations may predispose to early onset DCIS. However, the difference in the two odds ratios was not statistically significant, and other studies did not report association between *CHEK2* and early onset DCIS, so further studies are needed in this regard.

Our study suggests that the risk of DCIS in a woman with a *CHEK2* mutation is not determined solely by the presence of the mutation; penetrance is also dependent on her family history of cancer. We observed that the DCIS risk for a woman with a *CHEK2* mutation and a family history of breast cancer was greater (OR = 2.3, *p* = 0.003) than that of a *CHEK2* mutation carrier who has no family history of breast cancer (OR = 1.4, *p* = 0.06). Of note, the risk was highest for patients with a *CHEK2* truncating mutation and a positive family history of breast cancer (OR = 4.2, *p* = 0.01). These data support the model that both family history and a *CHEK2* mutation are risk factors for breast cancer that operate in concert, and that the *CHEK2* mutation modifies the risk of cancer imparted by other cancer genes [41–45].

We also analysed all cause survival of DCIS patients with a *CHEK2* mutation. Our data suggest the survival among *CHEK2* carriers with DCIS is good, and it is similar to that of non-carriers with DCIS. The 10-year mortality was 0% for carriers compared to 3% for non-carriers. Of note, Elshof et al. reported that 10-year breast cancer mortality among DCIS patients is 2.3% for women at age < 50 years, and 1.4% for those > 50 years at diagnosis [46]. According to Narod et al. at 20 years, the mortality among DCIS patients is 3.3% [47].

Other studies support association between *CHEK2* and DCIS [48–52]. The Breast Cancer Association Consortium reported the risk of 2.2 for DCIS in association with *CHEK2* truncating variants, 0.8% of 4187 DCIS patients carried a *CHEK2* truncating mutation [48]. Evans analyzed 311 women with DCIS, 4 (1.29%) had *CHEK2* c.1100delC mutation [49]. Petridis et al., studied DCIS patients diagnosed before age 50 and detected *CHEK2* pathogenic mutations in 2.4% of 655 DCIS cases, OR for a *CHEK2* mutation was 8.0 (95%CI 2.9–22.1) [50]. Mundt et al. found that *CHEK2* truncating variants confer 1.8-fold increased risk of DCIS, and *CHEK2* c.470T > C missense variant increases the risk by 1.3-fold (*p* = 0.02) [51]. Huang et al. showed that *CHEK2* truncating variants are associated with a moderate risk of DCIS (OR = 3.3, 95%CI 2.31–4.52) [52].

The aggregate evidence suggest that *PALB2* mutations may also predispose to DCIS, but this evidence is based on a few studies. Petridis et al. reported OR of 14.9 (95%CI 1.8–123.9) for DCIS given a *PALB2* mutation [50]. Huang et al. found that *PALB2* mutations confer 2.6-fold increased risk of DCIS (95%CI 1.26–5.12) [52]. The Breast Cancer Association Consortium also reported a moderate risk of 2.5 for DCIS in association with *PALB2* mutations [48]. We saw no association of *PALB2*, *NBN* and *RECQL* mutations with DCIS, but these mutations were rare in Polish women with DCIS, and our study is inconclusive in this regard. Other studies (than our study) have not investigated the role of *NBN* and *RECQL* in the etiology of DCIS.

In the current and our previous study [35] we described mutation frequencies in *BRCA1/2*, *PALB2*, *CHEK2*, *NBN* and *RECQL* in Polish women with DCIS. It is interesting to compare whether the mutation frequencies observed in our DCIS cohort differ from those in the general breast cancer population in Poland. For *BRCA1*, recurrent mutations were detected in 1.2% of DCIS cases compared to 3.6% in invasive breast cancer cases. This difference may be explained by the fact that *BRCA1* mutations predispose much strongly to invasive breast cancers than to DCIS [53]. They predispose in particular to medullar and ductal G3 tumors (usually without DCIS component), and *BRCA1*-associated breast cancers are commonly triple-negative, that is they have no expression of estrogen receptor (ER), progesterone receptor (PR) and human epidermal growth factor receptor 2 (HER2). Regarding *CHEK2*, truncating mutations were identified with similar frequency in DCIS cases (2.5%) and invasive tumors (2.9%). Also, *CHEK2* c.470T > C missense variant had similar prevalence (7.1%) in DCIS cases and invasive breast cancer cases (7.7%) [53]. These data suggest that *CHEK2* mutations confer similar risk of DCIS and invasive breast cancer. This is consistent with the evidence that breast tumors which arise in carriers of *CHEK2* mutations seem to be similar to those of breast cancers in the population at large. *PALB2* mutations were detected in 0.2% of DCIS cases compared to 0.9% of invasive breast cancer cases [14]. This suggests that *PALB2*-mutation carriers are more likely to be diagnosed with invasive breast cancer than with DCIS. For *NBN* and *RECQL*, the mutation frequencies were similar in DCIS cases and women with invasive breast cancer (0.7% vs. 1.0%, and 0.0% vs. 0.2%, respectively) [16, 54], and the number of mutation carriers was low (in DCIS), so no clear conclusions can be drawn. For *BRCA2*, we were not able to perform the comparison because *BRCA2* mutation frequency is not established in Polish women with unselected breast cancer. Of note, the above data are based on relatively low number of carriers among Polish DCIS cases in individual genes and further studies are needed.

The major reason why we focused our study on inherited predisposition to ductal carcinoma in situ is that women in Poland receive this specific diagnosis, and therefore we need to know whether or not DCIS may be caused by specific mutations in breast cancer susceptibility genes (and to what extent). This knowledge is important in clinical practice for several reasons. It provides useful information what mutations predispose to DCIS, and thus what genes should be screened in DCIS patients. Furthermore, if a mutation is found (i.e. in *BRCA1/2* or *CHEK2*) then specific surveillance protocols may be recommended for the mutation carriers (usually for breast and other cancers, because most breast cancer susceptibility genes are associated with a multi-site cancer predisposition). Finally, women with non-invasive breast tumors (in contrast to those with invasive cancers) are not commonly referred to outpatient genetic clinics in Poland [55]. Our evidence that mutations in *BRCA1/2* and *CHEK2* predispose Polish women to DCIS, should encourage clinicians to refer patients with DCIS for genetic counselling and testing more commonly.

## Conclusions

Based on the current study, women with a *CHEK2* mutation face an increased risk of DCIS, in particular in the presence of a positive family history of breast cancer. The presence of DCIS should be considered during surveillance of *CHEK2* mutation carriers, in particular when calcifications on mammography, a hypoechoic area on ultrasound or non-mass enhancement on breast MRI are seen. On the other hand, DCIS patients should receive genetic counseling and testing for *CHEK2* mutations.

## Data Availability

The datasets generated and/or analysed during the current study are not publicly available due the fact that the main research data supporting the results of this study are included in Tables 2 and 3 but are available from the corresponding author on reasonable request.

## Abbreviations

DCIS: Ductal carcinoma in situ
BC: Breast cancer
IBC: Invasive breast cancer
HBC: Hereditary breast cancer
OR: Odds ratio
*P*: Probability value
